# Placental pseudo-malignancy from a DNA methylation perspective: unanswered questions and future directions

**DOI:** 10.3389/fgene.2013.00285

**Published:** 2013-12-10

**Authors:** Boris Novakovic, Richard Saffery

**Affiliations:** ^1^Cancer and Disease Epigenetics, Murdoch Childrens Research Institute, Royal Children’s HospitalParkville, VIC, Australia; ^2^Department of Paediatrics, University of MelbourneParkville, VIC, Australia

**Keywords:** DNA methylation, trophoblasts, epigenomics, hypermethylation, global hypomethylation, cancer invasion, placental invasion

## Abstract

The growing fetus is dependent on adequate placental function for delivery of essential nutrients and oxygen, and for waste removal. The placenta also plays an important protective role; shielding the developing baby from the maternal immune system and adverse environmental exposures. Fundamental to these processes is correct invasion of the decidua and remodeling of maternal vasculature, each of which show remarkable parallels to tumorogenesis, with the obvious exception that the former is usually a tightly controlled process. It is not surprising that these physiological similarities are mirrored in gene expression and epigenetic parallels, many not found in any other aspect of human development. In this perspective, we summarize known DNA methylation similarities between placenta and human tumors, and discuss the implications and knowledge gaps associated with these findings. We also speculate on the potential origin of common DNA methylation features in these two disparate aspects of human physiology.

## MOLECULAR PATHWAYS SHARED BY PLACENTA AND CANCERS

Two of the most significant physiological similarities between the human placenta and cancer are invasion of surrounding tissue and evasion of the host immune response. In the placenta these tasks are primarily performed by the three distinct trophoblast populations; the stem-cell like villous cytotrophoblasts (VCT), invasive extravillous trophoblasts (EVT), and the syncytiotrophoblast (ST) layer. Invasion of decidua and remodeling of maternal vasculature is carried out by EVTs, enabling the supply of blood to the developing fetus via the placenta. Early *in vitro* studies of EVTs confirmed their invasive capacity (Yagel et al., 1988), with subsequent work showing the utility of transformed EVT cell lines as models for tumor progression (Lala et al., 2002). This work also highlighted the role of TGF-beta signaling via Smad3, and TIMP1 in the restriction of EVT invasion (Lala et al., 2002). Many other genes are implicated in the invasive properties of EVTs, several of which are also involved in tumor progression (reviewed extensively in Soundararajan and Rao, 2004; Pollheimer and Knofler, 2005; Ferretti et al., 2007).

Modulation of the maternal immune system is a major challenge for the developing pregnancy, a process that again displays features usually only seen in cancer (Mullen, 1998). Immune evasion is a multifaceted process that involves human chorionic gonadotrophin production by the ST layer, a lack of major histocompatibility complex class I and II antigen expression, selective expression of HLA-G, and evasion of TGF-beta (reviewed in Mullen, 1998; Holtan et al., 2009).

Other unique similarities between the placenta and cancer include expression of human telomerase (hTERT; Nishi et al., 2004), and placenta proteins (PPs) including SP1 (Inaba et al., 1980), PLAC1 (Devor and Leslie, 2013), and CGB/LHB [in breast cancer (Giovangrandi et al., 2001)]. Furthermore, a recent study of a panel of 293 lung tumors linked the expression of testis/placenta specific transcripts to lung cancer metastasis potential (Rousseaux et al., 2013). The expression similarities also include micro RNAs, with the 100 kb miRNA cluster, *C19MC*, exclusively expressed in human placenta and cancers in association with hypomethylation of an upstream CpG island (Bortolin-Cavaille et al., 2009; Tsai et al., 2009).

## GLOBAL HYPOMETHYLATION AND PARTIALLY METHYLATED DOMAINS ARE A HALLMARK OF CANCER AND THE PLACENTA

The earliest evidence for tumor-like methylome in the human placenta came from a study in 1983. Gama-Sosa et al. (1983) showed that the placental global methylation level (total % of 5-methylcytosine or 5meC) was approximately 3%, more similar to human tumors (2.5–3.5%) than disease-free somatic tissues (4–5%). Whereas a general increase in methylation occurs in most somatic cells in association with differentiation, this process appears attenuated in the extraembyronic lineage (Chapman et al., 1984), manifesting as a reduced level of methylation at specific repetitive DNA elements that comprises the bulk of mammalian genomic DNA (Tabano et al., 2010).

More recently, a substantial proportion of the placental genome (~37%) has been shown to comprise stretches (>100 kb) of low to intermediate methylation, generally highly methylated in somatic tissues. Such partially methylated domains (PMDs) have only previously been reported in some cancers and cell lines (e.g., IMR90 and SH-5Y5Y cells) (Lister et al., 2009; Schroeder et al., 2013). PMDs are associated with gene repression, and there is evidence that CpG Islands within PMDs are hypermethylated compared to CpG Islands within adjacent highly methylated domains (HMDs; Lister et al., 2009). The identification of PMDs in the placenta suggests that global hypomethylation in this tissue is not restricted to repetitive elements. Currently there is very little known about the establishment of PMDs and whether these regions are present in other cell types, although it is clear that many genes involved in placenta development and function (e.g., defense response) are found in placenta-specific HMDs (Schroeder et al., 2013). This is in line with a general higher expression of genes within HMDs, compared to PMDs, in the human placenta. The current view of PMDs in cancer is that they contribute significantly to the tumor specific hypomethylation. Further study of PMDs in placenta and cancer will provide more clues about the establishment, maintenance and function of global DNA methylation in placental development and disease.

At present it is unclear whether tumor-associated global hypomethylation is reflective of a reduced level of methylation in cancer precursor cells, or whether this is the result of a loss of methylation (either active or passive) as part of cancer progression. It is worth noting that even premalignant benign neoplasms are associated with a global decrease in DNA methylation (Goelz et al., 1985). The role of DNA methyltransferases in the genomic hypomethylation that is feature of cancer and the placenta remains unclear, as does the impact of this hypomethylation on genomic stability in the placenta; a hallmark of cancer-associated hypomethylation. We previously identified *DNMT1* (maintenance methyltransferase) promoter methylation in primate placenta (Novakovic et al., 2010), with subsequent studies demonstrating that *DNMT1* is imprinted specifically in placental tissue (Yuen et al., 2011; Das et al., 2013). However, the *DNMT1* promoter is not methylated in mouse placenta, suggesting it is not essential for global hypomethylation. A recent study in mice, showed that *in vitro* differentiation of ES cells to trophoblast results in downregulation of *Dnmt3a*2 and upregulation of *Dnmt3a1 de novo *methyltransferases, with no change in *Dnmt1* and *Dnmt3b* expression. Interestingly, Dnmt1 was shown not to localize to replication foci during S phase in this system in association with decreased expression of the Dnmt1 chaperone Np95 (Oda et al., 2013). It will be interesting to directly compare the temporal expression patterns of genes involved in DNA methylation/demethylation in both human extra-embryonic lineage and early malignancy.

## EPIGENETIC ACTIVATION OF RETROVIRAL PROMOTERS AND ENHANCERS

Transposable elements (TEs), including retrotransposons, comprise around half of the human genome. Some have demonstrated roles in gene regulation (Emera and Wagner, 2012). TEs can act as promoters, enhancers or insulators, and are believed to have contributed to the evolution of the placenta through the up-regulation of specific gene pathways, such as cAMP signaling, in placenta and endometrium (Lynch et al., 2011). Recent data have also identified a broader role for tissue-specific retroelement hypomethylation in association with enhancer activity (Xie et al., 2013) in humans.

Expression of genes through retrotransposon-derived promoters is uncommon in somatic human tissue, due to epigenetic silencing of these sequences (Emera and Wagner, 2012). However, in the placenta these elements drive the expression of several genes, including interleukin-2 receptor beta (*IL2RB*; Cohen et al., 2011). Furthermore, these elements can act as alternative promoters, giving rise to placenta-specific transcripts in genes such as *KCNH5, INSL4, EDNRB, PTN,* and *MID1* (Macaulay et al., 2011). In addition to activating existing genes, some previously “parasitic” elements such as Human Endogenous Retrovirus-encoded envelope proteins, Syncytin-1 and -2, EnvP(b), and EnvV, have become “domesticated” to perform essential functions in placentation (Gimenez et al., 2009; Vargas et al., 2009, 2012).

A recent transcriptome analysis of two placental and one non-placental mammal discovered that 40% of the placental-specific transposon family, *MER20* were in the proximity (<200 kb) of genes with endometrium-specific expression in placental mammals (Lynch et al., 2011). This analysis suggests that *MER20* acquisition by placental mammals played a major role in the evolution of the placenta. Placenta-specific retrotransposon-derived promoters can also be active in certain cancers in association with DNA hypomethylation. For example, the expression of *INSL4*, a candidate oncogene, is upregulated by hypomethylation of an LTR-derived promoter in placenta (Bieche et al., 2003) and thyroid and breast cancer (Brandt et al., 2005; Rodriguez-Rodero et al., 2013). Other placenta and cancer specific LTR promoter driven genes include envelope proteins, such as *ERVWE1*, which is hypomethylated in testicular cancer but not matched somatic tissue (Gimenez et al., 2010). At present, it remains unclear whether the global DNA hypomethylation seen in cancer results in a similar increase in the number of active retrotransposon promoters and enhancers elements (and associated changes in gene expression profile) as seen in the placenta. Further, it remains unclear why placental retrotransposon hypomethylation does not appear to be associated with active transposition of such elements in the genome, although few have investigated this in any detail. Finally, it is intriguing to speculate that placenta-specific epigenetic profiles of the different parasitic DNAs present in different species may play a role in the well documented differences in placental structure and function that are widespread in eutherians. This is supported by studies in the rat and mouse placenta that have identified epigenetic activation of rodent-specific endogenous retroviruses as a major driver of placental specific enhancer activity (Chuong et al., 2013).

## PLACENTA-SPECIFIC TUMOR SUPPRESSOR METHYLATION– A HIDDEN PATHWAY TO CANCER DEVELOPMENT?

Wide-spread DNA methylation-mediated silencing of tumor suppressor genes (TSGs) is a hallmark of human cancer (Timp and Feinberg, 2013). In the placenta, the search for specific DNA methylation biomarkers with utility for non-invasive diagnosis of pregnancy-associated disease, led to the discovery of placenta-specific hypermethylation of the *RASSF1A* TSG (Chan et al., 2006; Chiu et al., 2007). *RASSF1A* is involved in a variety of cellular pathways, and is the most commonly silenced TSG in human cancers (Agathanggelou et al., 2005) and the demonstration of placenta-specific methylation was the first identification of TSG silencing through hypermethylation in any non-cancerous tissue. This was closely followed by the report of Maspin silencing in first trimester placenta through histone repression (Dokras et al., 2006). Our group, and others, subsequently identified several TSGs with a placenta-specific methylation profile, most notably multiple negative regulators of canonical Wnt/ β-catenin signaling, including *APC, SFRP2, WIF1*, and *EN1* (Novakovic et al., 2008; Wong et al., 2008; Guilleret et al., 2009). This pathway plays a key role in cell migration and invasion associated with embryogenesis and tissue development via β-catenin regulation of gene expression (Polakis, 2012). There is now clear evidence for a role of Wnt signaling in placental function, specifically the differentiation of cytotrophoblasts to extravillous cytotrophoblasts (reviewed in Knofler and Pollheimer, 2013). Deregulation of this pathway is a key feature of many different cancers (Clevers, 2006), including placental related tumors and hydatidiform mole (Pollheimer et al., 2006).

There is a complex interplay between inhibition and promotion of Wnt signaling in the placenta which may play a role in controlled invasiveness. For example, the promoter of *WNT2* (ongogenic in cancer; Vider et al., 1996) shows placenta-specific hypermethylation (Yuen et al., 2009). In addition, the placenta shows an intermediate level of methylation at TSG promoters, as opposed to the complete hypermethylation observed in cancer. However, recent epigenomic analysis in cancers of different origins, suggests that most CpG Island hypermethylation events in cancer occurs at genes that are already silenced in the healthy tissue of origin (Sproul et al., 2012). Therefore, the majority of hypermethylation events may occur following cancer development and progression, and are therefore “passenger” methylation events (Sproul and Meehan, 2013; Timp and Feinberg, 2013). Nevertheless, there is evidence for a role of DNA methylation-mediated silencing in cancer progression and survival (De Carvalho et al., 2012). Whether the monoallelic methylation of TSGs in the human placenta plays a driving role in function and development is not certain, although the accumulation of β-catenin during trophoblast differentiation suggests that methylation of negative regulators of Wnt signaling has some functional role (Knofler and Pollheimer, 2013).

Unlike cancers, which have different origins, and therefore different “default” gene expression and methylation marks, methylation of TSGs in placenta appears to be an orchestrated developmental process, and in several cases may involve genomic imprinting (Guilleret et al., 2009). It would be very informative to pinpoint the exact time during development that these methylation marks are established, and if this results in an expression change. For example, are the marks present in the trophectoderm at the blastocyst stage? Unfortunately, it is not possible to measure methylation in primary human trophectoderm, and iPS cells have yet to be differentiated into trophectoderm. Therefore, we are limited to cells from the first trimester of pregnancy (~8 weeks), which already show mono-allelic methylation of TSGs. Furthermore, mouse studies are not useful, because the majority of the monoallelic methylation of TSGs observed in humans is not present in mice. However, trophoblastic choriocarcinoma cell lines give us some insights into the role of TSG methylation in placenta. In these cell lines, TSGs that show mono-allelic methylation in normal placenta are completely hypermethylated (Novakovic et al., 2008). This highlights the pre-malignant nature of normal human placentation, in that the TSG methylation is developmentally established to the extent that it facilitates normal development, but prevents overt invasion of trophoblasts. Therefore, the placenta displays a limited profile of TSG methylation, which may play a key role in controlling the “pseudomalignant” nature of placentation.

## CANCER ASSOCIATED DNA METHYLATION AND TROPHOBLAST FUNCTION

The coordinated, placenta-specific methylation of several TSGs constitutes *prima facie* evidence for a role of DNA methylation in regulating trophoblast function. However, in many instances this has not yet been directly demonstrated, primarily due to the lack of suitable model systems. Commonly used cell lines are often derived from placental malignancies whereas non-primates placentae lack much of the observed TSG methylation seen in primates (Ng et al., 2010). The most informative data will likely come from studies in isolated primary trophoblasts. One such study has linked higher methylation at two TSGs, *MMP2*, and *PRKCDBP*, with trophoblast invasion (van Dijk et al., 2012). A systematic and expanded analysis of this kind has the potential to identify many epigenetically regulated genes with essential roles in trophoblast function. An alternative approach is to link observational studies in humans to functional analysis in cell lines. For example, Shi et al. (2012) identified high MASPIN expression in a cohort of women with preeclampsia, and using 5-aza treatment (a DNA methylation inhibitor) showed that MASPIN demethylation, and elevated expression, decreased proliferation and migration in the TEV1 trophoblastic model cell line.

## LOSS OF IMPRINTING IN HUMAN PLACENTA

Loss of imprinting (LOI) refers to the gain of expression from the usually silenced allele of an imprinted gene (Lambertini et al., 2008b). LOI is one of the earliest and most common epigenetic aberrations in cancers (Jelinic and Shaw, 2007). A number of studies have demonstrated that LOI occurs frequently in human term placenta, with much higher rates in the first trimester (Lambertini et al., 2008a; Diplas et al., 2009b). The authors argued that LOI in the first trimester might be a regulatory mechanism promoting trophoblast invasion and the establishment of placentation. Furthermore, the lack of correlation between LOI and gene expression level, and the all-or-nothing pattern of LOI at the single cell level in cytotrophoblasts, suggest that LOI plays a role in placental development by introducing variation in cell phenotype (Diplas et al., 2009a; Pozharny et al., 2010).

It is possible that frequent LOI in human placenta is due to DNA-methylation-mediated silencing of *DNMT1* (possibly maternally imprinted) in primate placenta (Yuen et al., 2011; Das et al., 2013). Potentially, *DNMT1* imprinting may result in less strict maintenance of mono-allelic methylation at several imprinted genes. A case for a role of DNMT1 in controlling DNA methylation at imprinted genes comes from the observation that *DNMT1* is completely hypomethylated in trophoblastic choricarcinoma cell lines, while TSGs with monoallelic methylation in normal placenta are completely hypermethylated in these cell lines (Novakovic et al., 2008). Furthermore, LOI is not common in the mouse placenta, which potentially also has more genes under imprinting control. This has led to suggestions that genomic imprinting is more important in mice compared to humans, due to lack of intra-uterine competition in the latter (Monk et al., 2006). Interestingly, the mouse placenta is less invasive, with little similarity to an invasive tumor, and the *Dnmt1* promoter is not methylated (Ng et al., 2010). It would therefore be interesting to test if LOI is common in baboon and marmoset placenta, which are also more invasive, and in which the *DNMT1* promoter is mono-allelically methylated (Novakovic et al., 2010). A final note on the role of methylation-mediated DNMT1 imprinting would be that in our previous comparative analysis, we did not identify an association between DNMT1 methylation and global hypomethylation in the placenta. Indeed, both primates and non-primate placentas showed hypomethylation, regardless of DNMT1 promoter methylation level (Novakovic et al., 2010). Therefore, the potential role for *DNMT1* imprinting in the frequent LOI or any other epigenetic phenomenon in the placenta needs to be investigated further.

## A COMMON ORIGIN OF DNA METHYLATION FEATURES IN THE PLACENTA AND CANCER?

Identified epigenetic similarities conserved in placentation and cancer span a broad range of processes and cellular pathways, from an overall global hypomethylation of genomic DNA, conserved across species, to a specific profile of Tumor Suppressor gene methylation, largely restricted to primates (**Table 1**). Such coordinated epigenetic features may simply reflect the independent use of the same similar pathways for cell invasion, immune modulation and vascular remodeling associated with the rapid evolution of this tissue across mammals. More intriguingly, the higher incidence of cancer in placental mammals relative to other lineages raises the possibility that cancer may involve inappropriate “reactivation” of molecular pathways usually restricted to placental development.**Either way, it is now clear that studying epigenetics in the placenta offers a unique opportunity to understand the processes leading to aberrant epigenetic change associate with human cancer. However, this will not be easy given the previously described lack of conservation of many epigenetic features of primate placentas, and the clear temporal changes in cell composition and epigenetic status in human placentation throughout pregnancy. Further, the placenta is a complex tissue made up of multiple cell types each with a specific function. Each needs to be examined as purified populations, preferably over the length of gestation. Only then will it be possible to link specific tumor-like epigenetic marks to specific placental cell subtypes and cell functions.

**Table 1:** Key epigenetic similarities between the placenta and human cancer.

Feature	Detail	
Global hypomethylation	Placental tissue and human cancers show a global 5-methylcytosine content of ~3%, lower than that of all disease-free somatic tissues (4–5%). This is associated with a loss of methylation at specific repeat sequences (mainly LINE1) that comprise the bulk (~50%) of genomic DNA and long genomic regions of intermediate methylation, called partially methylated domains (PMDs), which cover 37% of the placental genome. The presence of PMDs is a feature of most human cancers.	Gama-Sosa etal. (1983), Tabano etal. (2010), and Schroeder etal. (2013)
Tumor suppressor gene (TSG) methylation	*RASSF1A* is hypermethylated in almost every cancer type and was the first TSG shown to be specifically methylated in the placenta. Methylation of multiple negative regulators of Wnt signaling (APC, WIF-1, SFRP2, EN1) demonstrates coordinated epigenetic regulation of signaling pathways in placenta and cancer. Multiple examples of monoallelic methylation indicate a potential suite of novel imprinted genes involved in the regulation of placentation in humans.	Chiu etal. (2007), Novakovic etal. (2008), Wong etal. (2008), and Guilleret etal. (2009)
Loss of imprinting (LOI)	Imprinting (parent of origin allele specific expression) is regulated by DNA methylation. LOI common in placenta, and is one of the most consistent epigenetic aberrations observed in cancer.	Lambertini etal. (2008a) and Diplas etal. (2009b)
Cryptic promoter/enhancer activation	Global hypomethylation results in placenta-specific activation of transposable elements to produce novel promoter and enhancer elements. This leads to generation of placenta-specific transcripts of endogenous genes. Additionally, several retrotransposable elements are exclusively expressed in the placenta and cancer and are essential for faithful placentation.	Macaulay etal. (2011) and Vargas etal. (2012)
miRNAs	Some miRNA genes, including the large microRNA cluster C19MC, are expressed only in the placenta and in human cancers in association with hypomethylation of an upstream CpG islands. Altered miRNA expression leads to placental/cancer specific changes in downstream gene expression of target loci.	Bortolin-Cavaille etal. (2009)

## A ROADMAP FOR FUTURE EPIGENETIC STUDIES IN THE PLACENTA

The majority of studies aimed at identifying gene expression or epigenetic aberrations associated with pregnancy outcome or later health have been done on whole placental biopsies collected at delivery. This is inappropriate for two reasons: firstly, the placenta is a complex tissue made up of numerous cell types, each known to show gene expression and epigenetic differences. Analysis of whole tissue is likely to mask molecular features confined to specific cell types, which is also a problem in cancer studies, as cancer biopsies contain several different cell types. Further, the full term placenta is morphologically and functionally distinct from the early pregnancy placenta, a likely critical time point for the development of pregnancy-associated problems. Thus an analysis of first trimester samples (obtainable from elective termination or as part of chorionic villi sampling (CVS) is essential, as is purification of the multiple different cell types that comprise this tissue. Such an approach will most likely require refinement of current methodologies for cell isolation from placental biopsies and the identification of additional cell type-specific cell surface markers for cell sorting. CVS collection also provides a unique opportunity to perform functional studies on first trimester placental cells in culture (Campbell et al., 2007) although it is clear that the trophoblast compartment is not the predominant cell population in CVS cultured tissue. The integration of *in vitro* studies, epigenetic profiling and pregnancy outcome at term, will provide important links between placental function and outcome. Another important area of future study will be the elucidation of mechanism behind DNA methylation and demethylation during trophoblastic differentiation and development. State-of-the-art-technological advances can now be used to measure several different forms of methylated cytosine, including 5-hydroxymethylation (5hmC), 5-formylcytosine (5fC), and 5-carboxylcytosine (5caC). Generally present at lower levels than 5MeC, these forms of methylated cytosine are likely intermediates in a demethylation process, but may also have specific functional roles in gene expression regulation. Profiling such modifications in the placental may therefore provide valuable insight into specific aspects of placental function and/or spatial and temporal control of DNA methylation maintenance and removal in the human placenta. It will be especially interesting to determine the role of these modifications during the differentiation process from stem cell like VCTs to EVTs and the ST layer. Specifically, whether some genes are subject to active demethylation during this process. Finally, it is likely that many of the differences seen in mammalian placentas (the most rapidly evolving tissue/organ in mammals) are specified, at least in part, epigenetically. Therefore, comparing epigenetic profile across species represents a valuable approach for identifying epigenetic features underlying key processes specific to primate placentation.

## Conflict of Interest Statement

The authors declare that the research was conducted in the absence of any commercial or financial relationships that could be construed as a potential conflict of interest.
